# The Infancy of Christ

**Published:** 1890-01-25

**Authors:** 


### Words of Consolation.

### THE INFANCY OF CHRIST.

*(For Reading to the Sick.)*

We are all greatly interested and attracted by reading accounts of the infancy and youth of great men, partly, perhaps, because it makes them appear nearer our own level. For how wondrous strange it seems, when we reflect on it, that the greatest conqueror, the most powerful monarch, the ablest statesman, the wisest philosopher, have, like ourselves, passed through the puling age of infancy, and been "under tutors and governors until the time appointed by the Father." They were all under subjection and learned to obey in youth, and hence came their power to rule in manhood. But besides this, they have all gone through the stage of being guarded and governed by women only. It is the loving mother or nurse who has tended their years of helplessness, and whose delight was in watching the infant's opening charms. She it was who bathed daily the little form, who noted its developing strength and beauty, who incited it to laughter that she might enjoy its smiles and dimples, and, crumpling up the sweet face with her hand, would cover it thick with kisses. It was she who dared to correct its little faults and tempers, and dry its tears of sorrow, and, as days went by, guided the tottering footsteps and taught the little one to lisp its earliest prayers at her knee. What a happy time for the mother to look back on! Ah! yes, the greatest men who ever lived have been happy innocent children once—happy, too, for them if they keep the child-like heart through the heat and burden of life's long day.

We turn naturally at this season to the greatest man that ever lived, "even the Man Christ Jesus," and remember that He was no exception to the rule of humanity. The events of our Lord's life, for the first thirty years, that is, from His birth to the beginning of His ministry, have been veiled from us, but we may be permitted to dwell, with all reverence, on the privileges extended to the Virgin Mary during that time. She must have experienced all, and more than all, the usual joys of maternity, and rejoiced in the intense happiness which came from being the mother of a sinless Son.

She had sorrows, too, many and great. Who can picture the fear and agony of her heart when, warned of God in a dream, Joseph fled with her and the Holy Babe into Egypt to escape the furious rage of the cruel Herod? The two years' sojourn in that hostile and idolatrous land were necessarily full of care and anxiety, and her happiness only began when, led by a vision from God, the little family returned into the land of Israel and settled in the beautiful village of Nazareth. During this time of quiet and retreat, we are told that the "Child Jesus grew in wisdom and stature, and in favour with God and man," and that "He was subject to His parents." Happy parents! happy Child! to live in this threefold tie of love.

Every year the little family went up to Jerusalem to the feast of the Passover, and at one of these visits, when our Lord was twelve years old, occurred His mysterious loss. How, even for a moment, His mother forgot her great treasure, we know not, but the passing carelessness was dearly paid for by the three days of anxiety and sorrow which they passed before they found Him in the Temple. The gentle expostulation of the mother, "Son, why hast Thou so dealt with us?" only received for answer, "Wist ye not that I must be about My Father's business?" If Mary had forgotten for what purpose her Son had been sent into the world, the lesson she received was permanent, for "she kept all these things in her heart." How many of us, like Mary, forget our treasures are not our own. We grudge our children to the mission field where they are about their Father's business, spreading His light around; we grudge our money being used for charitable purposes. Our health is bad, our talents are not appreciated, all we cared for is passing from us. Happy for us if we bear these shocks and rebukes humbly, saying only: We have sought Thee, sorrowing. Yea, Lord, we own our negligence in using our gifts to Thy glory, our selfishness in keeping our best from Thy service. Lead us back, we beseech Thee, till we find Him whose meat and drink was to do His Father's will, and grant that we may be one with Him even as He is One with Thee, God, for ever and ever. Amen.

